# Supplementary material for: Healthy-like CD4+ Regulatory and CD4+ Conventional T-Cell Receptor Repertoires Predict Protection from GVHD Following Donor Lymphocyte Infusion
Source: Int J Mol Sci. 2022 Sep 18;23(18):10914. doi: 10.3390/ijms231810914 (PMC9505302; doi:10.3390/ijms231810914)
Supplement: Supplementary file 1 [file ijms-23-10914-s001.zip › ijms-1901889-supplementary.pdf]

Supplementary Materials

Supplementary Figure S1. GVHD post DLI is associated with reduced CIR and increased NRM.

Supplementary Figure S2. Clonal space and repertoire proportions GVHD vs. HC.

Supplementary Figure S3. Diversity in steroid samples over time.

Supplementary Figure S4. Future GVHD post DLI is not reflected in $CD4^+CD25^+CD127^{low}$ T_{reg} and $CD4^+$ T_{con} repertoire prior to DLI.

Supplementary Figure S5. Future GVHD post DLI is not reflected in $CD4^+CD25^+CD127^{low}$ T_{reg} and $CD4^+$ T_{con} repertoire of the DLI cell product.

Supplementary Figure S6. Gating strategy for cell sorting.

Supplementary Table S1. Disease and transplant characteristics.

Supplementary Table S2. DLI characteristics.

Supplementary Table S3. Healthy controls' characteristics.

Supplementary Table S4. Details about GVHD post DLI.

Supplementary Table S5. Correlation analyses between absolute cell counts and 1/D.

Supplementary Table S6. Clinical characteristics of expanded and not expanded samples at first time point post DLI.

Supplementary Table S7. Patient samples.

Supplementary Table S8. Healthy control samples.

Supplementary Table S9. Antibody panel for flow cytometry.

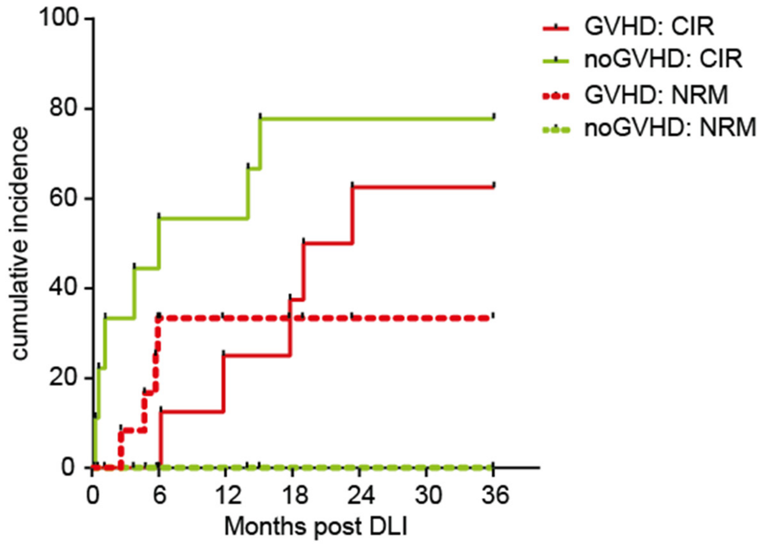

Number at risk:

GVHD	12	12	7	6	4	4	3
noGVHD	9	5	5	3	3	3	2

Supplementary Figure S1. GVHD post DLI is associated with reduced CIR and increased NRM. Cumulative incidence curves are displayed with relapse incidence (CIR, solid lines) and non-relapse mortality (NRM, dotted lines) as competing events for patients with GVHD (red) vs. patients without GVHD (green) within the 36 months study follow-up (CIR, P=0.024; NRM, P=0.067). CIR and NRM were assessed by means of cumulative incidence curves by Gray's test.

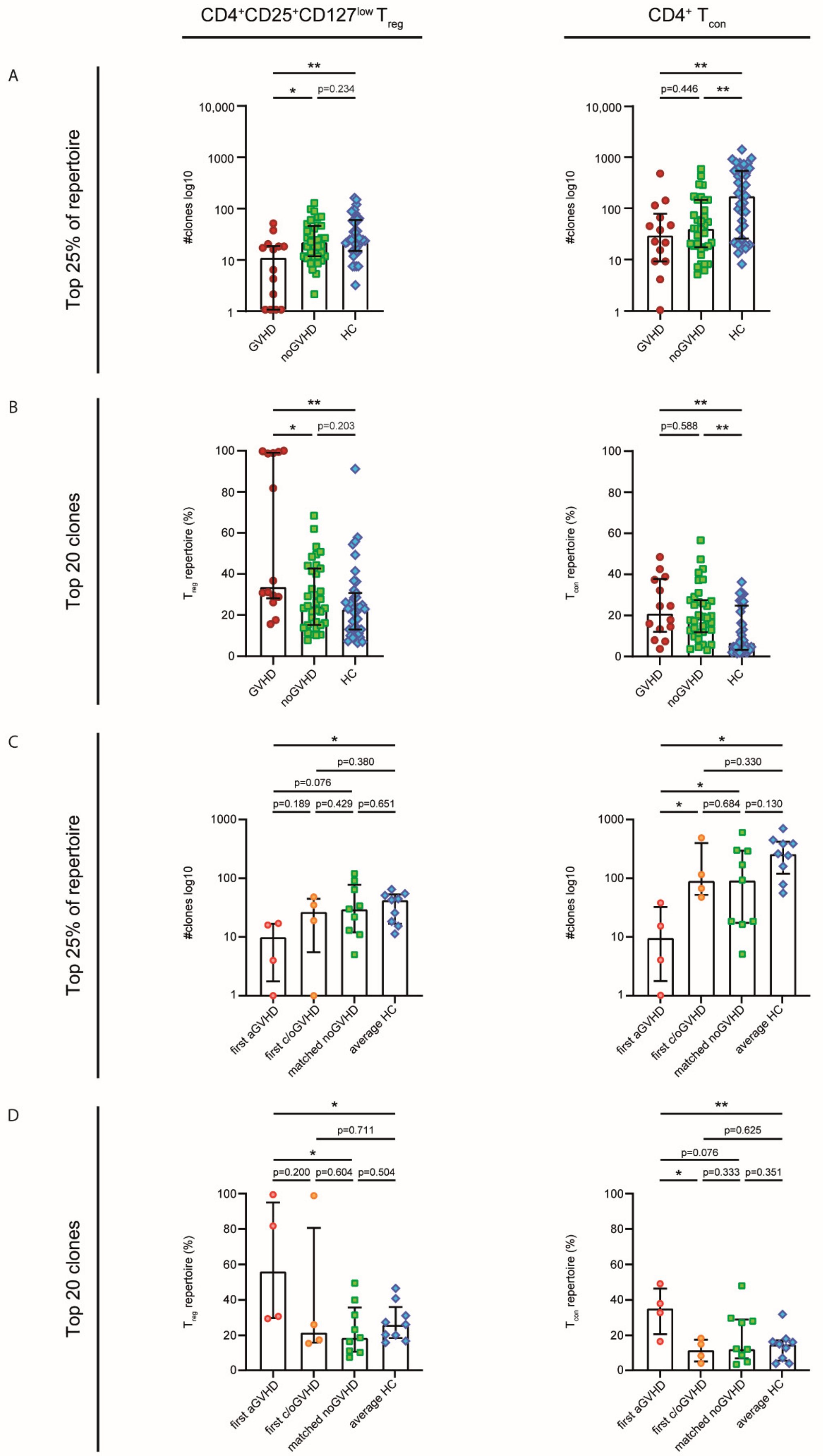

CD4+CD25+CD127low Treg
CD4+ Tcon
A
Top 25% of repertoire
#clones log10
GVHD
noGVHD
HC
p=0.234
p=0.446
B
Top 20 clones
Treg repertoire (%)
Tcon repertoire (%)
p=0.203
p=0.588
C
Top 25% of repertoire
first aGVHD
first c/oGVHD
matched noGVHD
average HC
p=0.380
p=0.076
p=0.189
p=0.429
p=0.651
p=0.330
p=0.684
p=0.130
D
Top 20 clones
p=0.711
p=0.200
p=0.604
p=0.504
p=0.625
p=0.076
p=0.333
p=0.351

Supplementary Figure S2. Clonal space and repertoire proportions GVHD vs. HC. (**A, B**) Comparison of both $CD4^+CD25^+CD127^{low}$ T_{reg} and $CD4^+$ T_{con} repertoire clonality in all patients' samples with GVHD post DLI (dark red circles, n=14) or without GVHD (noGVHD, green square, n=35) compared to healthy controls (HC, blue rhomb, n=39). (**A**) Number of unique clonotypes (y axis) required to occupy 25% of the $CD4^+CD25^+CD127^{low}$ T_{reg} (left panel) and the $CD4^+$ T_{con} repertoire (right panel). (**B**) Clonal proportions of the $CD4^+CD25^+CD127^{low}$ T_{reg} (left panel) and $CD4^+$ T_{con} repertoire (right panel) for the top 20 clones. (**C, D**) Comparison of samples with first occurrence of acute (a)GVHD (light red circle, n=4) and chronic or overlapping (c/o)GVHD (orange circle, n=4), samples without GVHD (matched for timing, n=9) and average clonality in HC (n=9). (**C**) Number of unique clonotypes (y axis) required to occupy 25% of the $CD4^+CD25^+CD127^{low}$ T_{reg} (left panel) and the $CD4^+$ T_{con} repertoire (right panel). (**D**) Clonal proportions of the $CD4^+CD25^+CD127^{low}$ T_{reg} (left panel) and $CD4^+$ T_{con} repertoire (right panel) for the top 20 clones. Two-tailed Mann-Whitney test or unpaired Student t-test was used for inter-group comparisons. Black lines indicate median, error bars show the interquartile range. ** $p<0.01$; * $p<0.05$.

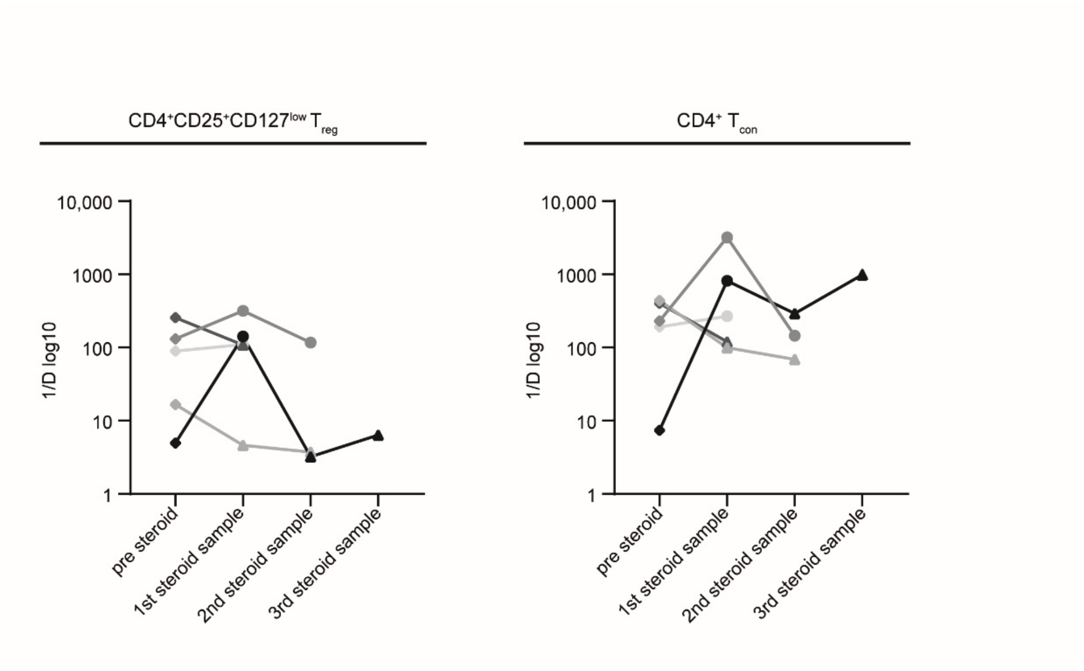

Supplementary Figure S3. Diversity in steroid samples over time. The graphs show both $CD4^{+}CD25^{+}CD127^{low}$ T_{reg} (left panel) and $CD4^{+}$ T_{con} repertoire (right panel) diversity in patients receiving treatment with systemic steroids (>7.5mg, n=5) over time. Only patients with available pre-steroid samples were included. Each line illustrates the repertoire dynamics of an individual patient, with matching gray shades in the left and right panel. Rhombs display samples prior to steroid treatment. Circles represent steroid sensitive samples (n=4), triangles represent steroid refractory samples (n=5).

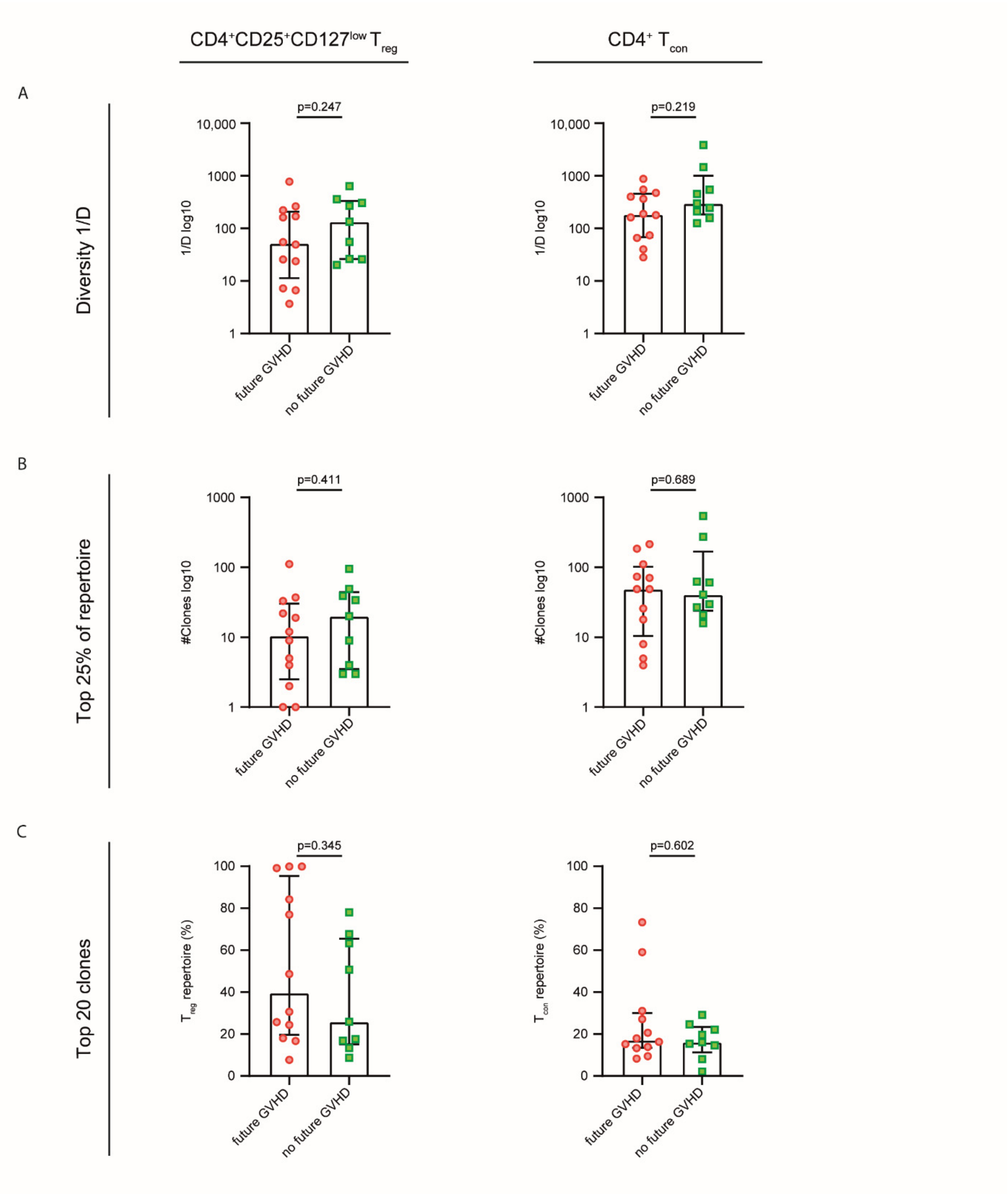

Supplementary Figure S4. Future GVHD post DLI is not reflected in $CD4^+CD25^+CD127^{low}$ T_{reg} and $CD4^+$ T_{con} repertoire prior to DLI. (**A, B, C**) The $CD4^+CD25^+CD127^{low}$ T_{reg} (left panel) and $CD4^+$ T_{con} repertoire (right panel) is compared between patient samples prior to DLI with future development of GVHD (red circle, n=12) during the 36 months study follow-up and patient samples prior to DLI without future

development of GVHD (green square, n=9). (**A**) shows the diversity of $CD4^{+}CD25^{+}CD127^{low}$ T_{reg} and $CD4^{+}$ T_{con} repertoire. (**B**) Comparison of the number of unique clonotypes (y axis) required to occupy the top 25% of the $CD4^{+}CD25^{+}CD127^{low}$ T_{reg} and $CD4^{+}$ T_{con} repertoire. (**C**) The graphs display the $CD4^{+}CD25^{+}CD127^{low}$ T_{reg} and $CD4^{+}$ T_{con} repertoire occupied by the top 20 clones. Two-tailed Mann-Whitney test or unpaired Student t-test was used for inter-group comparisons. Black lines indicate median, error bars show the interquartile range. ** $p<0.01$; * $p<0.05$.

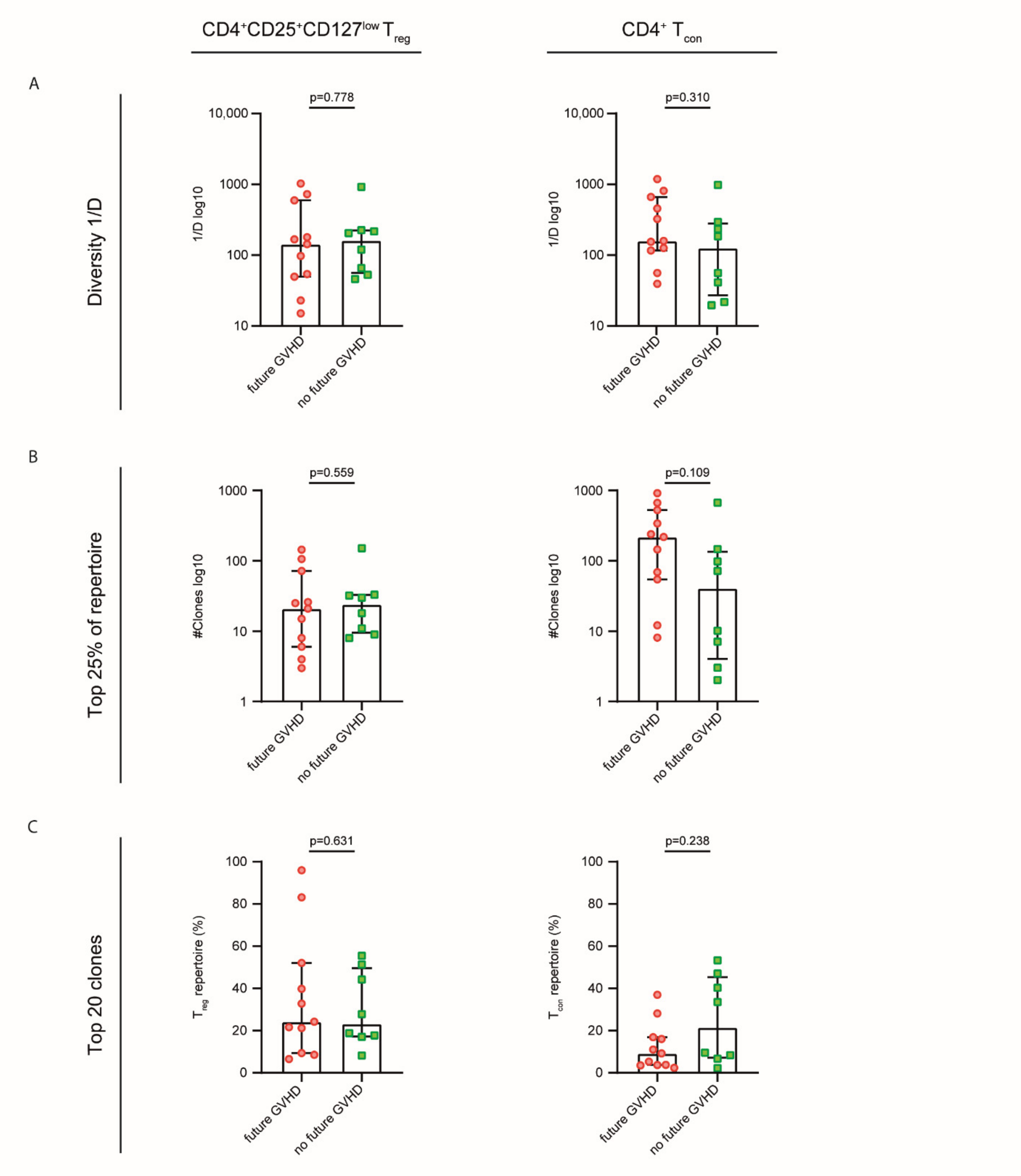

Supplementary Figure S5. Future GVHD post DLI is not reflected in $CD4^+CD25^+CD127^{low}$ T_{reg} and $CD4^+$ T_{con} repertoire of the DLI cell product. (**A, B, C**) The $CD4^+CD25^+CD127^{low}$ T_{reg} (left panel) and $CD4^+$ T_{con} repertoire (right panel) is compared between DLI product samples of patients with future development of GVHD (red circle, n=11) during the 36 months study follow-up and DLI product samples of patients without future development of GVHD (green square, n=8). Patient 018 and 027 were excluded from this

analysis since there were only donor's peripheral blood samples available. (**A**) Comparison of the diversity 1/D of $CD4^+CD25^+CD127^{low}$ T_{reg} and $CD4^+$ T_{con} repertoire. (**B**) Comparison of the number of unique clonotypes (y axis) required to occupy the top 25% of the $CD4^+CD25^+CD127^{low}$ T_{reg} and $CD4^+$ T_{con} repertoire. (**C**) The graphs display the $CD4^+CD25^+CD127^{low}$ T_{reg} and $CD4^+$ T_{con} repertoire occupied by the top 20 clones. Two-tailed Mann-Whitney test or unpaired Student t-test was used for inter-group comparisons. Black lines indicate median, error bars show the interquartile range. ** $p<0.01$; * $p<0.05$.

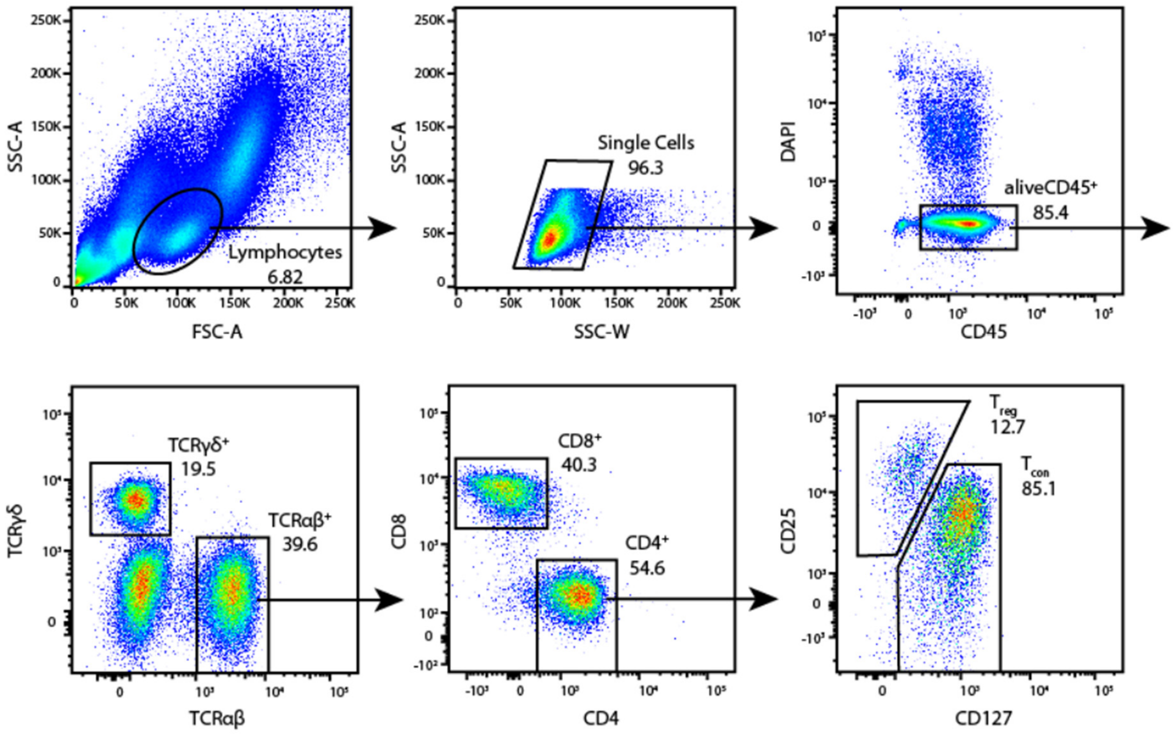

Supplementary Figure S6. Gating strategy for cell sorting.

Supplementary Table S1. Disease and transplant characteristics.

Abbreviations: AA, aplastic anemia; alloHSCT, allogeneic hematopoietic stem cell transplantation; AML, acute myeloid leukemia; BM, bone marrow; c-ALL, common acute lymphatic leukemia; DLI, donor lymphocyte infusion; ELN, European Leukemia Net; ET, essential thrombocythemia; f, female; IPSS-R, revised international prognostic scoring system; m, male; MDS, myelodysplastic syndrome; MPN, myeloproliferative neoplasm; PBSC, peripheral blood stem cells; PMF, primary myelofibrosis; PV, polycythemia vera; sAML, secondary acute myeloid leukemia; SMF, secondary myelofibrosis; tAML, therapy-associated acute myeloid leukemia. *Disease risk according to ELN [26] and IPSS-R [27].

Patient ID	Patient sex	Age at first DLI (years)	Disease details	Disease risk according to ELN and IPSS-R*	Donor sex	Donor age at alloHSCT (years)	Graft source
01	f	36	AML	intermediate	f	43	PBSC
02	m	63	MDS	high	m	22	PBSC
03	f	23	c-ALL	standard	m	29	PBSC
07	f	55	PMF/MDS-MPN	intermediate	m	57	PBSC
08	f	57	MDS	intermediate	m	58	PBSC
09	m	72	sAML from MDS	intermediate	m	22	PBSC
10	f	39	tAML	adverse	m	30	PBSC
11	m	45	AML	intermediate	f	45	PBSC+BM
12	f	50	AML	intermediate	f	26	PBSC
13	f	64	sAML from MDS	intermediate	f	64	PBSC
16	f	36	sAML from MPN	intermediate	m	31	PBSC
17	f	48	ET/SMF	-	m	31	PBSC
18	m	36	AML	adverse	f	27	BM
19	f	51	AML	intermediate	f	45	PBSC
20	m	55	PV	-	m	26	PBSC
22	m	50	AML	favorable	m	51	PBSC
23	m	65	AML	adverse	m	61	PBSC
25	m	41	AML	intermediate	m	29	PBSC
27	f	39	sAML from AA	intermediate	f	35	BM
30	m	40	tAML	adverse	m	27	PBSC
33	f	33	AML	adverse	f	30	PBSC

Supplementary Table S2. DLI characteristics.

Abbreviations: alloHSCT, allogeneic hematopoietic stem cell transplantation; BW, body weight; CR, complete remission; CR_i, CR with incomplete hematologic recovery; CTx, chemotherapy; DAC, Daunorubicin + Cytarabine + Cladribine; DLI, donor lymphocyte infusion; EM, extramedullary; FLA-IDA, Fludarabine + Cytarabine + Idarubicin; Gy, Gray; hr, high risk; iHC, increased host chimerism; PD, progressive disease; R, Relapse; RD, resistant disease; RTx, Radiatio (dose in Gy); SAIL, Selinexor + Cytarabine + Idarubicin; V-I-PEG-A-Dexa, Vindesine + Idarubicin + PEG-Asparaginase + Dexamethasone. *according to ELN response criteria [26].

Patient ID	DLI Trigger	CTx pre DLI	Months post CTx at first DLI	Response to CTx*	Months post alloHSCT at first DLI	Dose first DLI ($CD3^+$/ kg BW)	Total number of DLI	Cumulative dose DLI ($CD3^+$/kg BW)
01	Cytologic R	SAIL	1.4	CR_i, iHC	7.9	$1x10^7$	1	$1\ x10^7$
02	Cytologic R	FLA-IDA	1.4	PR	23.8	$1.2x10^7$	1	$1.2\ x10^7$
03	Cytologic R	V-I-PEG-A-Dexa	1.6	RD	8.8	$1x10^7$	1	$1\ x10^7$
07	iHC	-	-	-	10.3	$0.1x10^7$	3	$0.7\ x10^7$
08	iHC	-	-	-	10.5	$0.1x10^7$	2	$0.6\ x10^7$
09	Cytologic R	Decitabine	0.2	PR	29.4	$1x10^7$	1	$1\ x10^7$
10	Cytologic R	Azacitidine, Sorafenib	0.3	PR	5.6	$0.5x10^7$	1	$0.5\ x10^7$
11	hr Genotype	-	-	-	9.2	$0.1x10^7$	4	$6.6\ x10^7$
12	Histologic R	-	-	-	17.1	$1x10^7$	3	$16\ x10^7$
13	iHC	-	-	-	8.1	$0.1x10^7$	4	$5.6\ x10^7$
16	Histologic R	SAIL	1.9	CR_i, iHC	22.0	$0.5x10^7$	7	$32.5\ x10^7$
17	Histologic R	-	-	-	24.0	$0.1x10^7$	3	$1.1\ x10^7$
18	Molecular R	-	-	-	9.6	$0.5x10^7$	1	$0.5\ x10^7$
19	hRG	-	-	-	8.3	$0.1x10^7$	2	$0.6\ x10^7$
20	Molecular R	-	-	-	9.3	$0.1x10^7$	4	$3.4\ x10^7$
22	Cytologic R	FLA-IDA	1.2	PR	14.2	$1x10^7$	2	$6\ x10^7$
23	Cytologic R	Azacitidine	0.3	PR	4.6	$0.5x10^7$	1	$0.5\ x10^7$
25	Cytologic R	SAIL	0.9	CR, iHC	75.7	$1x10^7$	1	$1\ x10^7$
27	iHC	-	-	-	11.0	$2.5x10^7$	3	$11.5\ x10^7$
30	Molecular R	FLA-IDA	1.4	CR, iHC	26.3	$1x10^7$	2	$6\ x10^7$
33	Cytologic R + EM R	DAC	1.1	CR, iHC	11.5	$1x10^7$	1	$1\ x10^7$

Supplementary Table S3. Healthy controls' characteristics.

Abbreviations: ALC, absolute lymphocyte count; f, female; Hb, hemoglobin; HC, healthy controls; m, male; PLT, platelets; WBC, white blood cell count.

HC ID	Age (years)	Sex	CMV IgM/IgG	EBV IgM/IgG	WBC, average (/µl)	ALC, average (/µl)	Hb, average (g/dl)	PLT, average (/µl)
H16.02	46	m	−/+	−/+	4920	1770	13.9	332,000
H16.03	58	m	+/+	+/+	6600	1860	15.1	257,330
H16.04	66	m	−/+	−/+	8320	1940	16.1	235,670
H16.06	28	m	−/+	−/+	7950	3810	13.9	287,000
H16.07	21	m	−/−	−/−	7780	2410	14.2	347,670
H16.11	33	f	−/−	−/+	7890	1800	13.3	343,330
H16.12	43	f	−/−	−/−	4170	1650	15.2	179,330
H16.13	53	f	−/−	−/+	8230	1350	14.6	215,670
H16.14	31	f	−/+	−/+	6690	2540	12.1	246,670

Supplementary Table S4. Details about GVHD post DLI.

Abbreviations: aGVHD, acute graft-versus-host disease; alloHSCT, allogeneic hematopoietic stem cell transplantation; c/oGVHD, chronic/overlap graft-versus-host disease; DLI, donor lymphocyte infusion; GI, gastrointestinal tract; MMF, mycophenolate mofetil; IS, immunosuppression. *according to MAGIC criteria [34]; **according to NIH 2014 criteria [35]; ***according to EBMT-NIH-CIBMTR task force panel recommendation [31].

Patient ID	First GVHD, months post DLI	aGVHD post DLI: grade*; organ involvement	c/oGVHD post DLI: grade**; organ involvement	Treatment with systemic steroids >7.5mg	Response to steroids***	Additional IS	Relapse post DLI, months
01	0.6	III; skin, GI, liver	-	yes	sensitive	-	-
02	1.1	III; skin, GI, liver	-	yes	sensitive	-	-
08	15.4	-	mild; eyes, mucosa	no	-	-	-
10	1.3	IV; skin, GI, liver	severe; GI, liver, eyes, mucosa	yes	refractory	MMF, infliximab, sirolimus	17.7
11	3.2	II; liver	moderate; skin, liver, eyes, mucosa	yes	sensitive	-	11.3
12	5.1	II; skin, GI	severe; skin, GI, liver, mucosa, eyes, joints	yes	sensitive + refractory	-	19.3
16	3.1	I; skin	-	no	-	-	6.1
17	3.2	IV; skin, liver	severe; skin, liver, eyes, mucosa	yes	refractory	MMF	-
20	7.8	I; skin	moderate; skin, eyes, mucosa	yes	sensitive	-	-
22	2.3	IV; skin, liver	severe; skin, liver, eyes, mucosa	yes	refractory	MMF	-
27	14.4	III; skin, GI	-	no	-	-	11.7
30	1.2	I; skin	severe; skin, liver, lung, eyes, mucosa	yes	sensitive + refractory	MMF	-

Table S5. Correlational analyses between absolute cell counts and 1/D.

Correlation between absolute cell counts and diversity in GVHD and no GVHD patients as well as healthy controls was tested by Pearson correlation coefficient. Abbreviations: GVHD, graft-versus-host disease; HC, healthy controls; r, correlation coefficient

	Population	Pearson r	P value
$CD4^+CD25^+CD127^{low}$ T_{reg}	GVHD	0.054	0.860
	noGVHD	0.103	0.556
	HC	−0.023	0.892
$CD4^+$ T_{con}	GVHD	−0.295	0.328
	noGVHD	0.122	0.486
	HC	0.285	0.079

Table S6. Clinical characteristics of expanded and not expanded samples at first time point post DLI.

Shown is the absolute number (%) for categorical variables and the median absolute value (range) for numerical variables. Fisher's exact test (two-tailed) was used for comparison of categorical variables; numerical variables were analyzed with unpaired Student t-test (normally distributed data) or two-tailed Mann-Whitney test (not normally distributed data). Abbreviations: aGVHD, acute graft-versus-host disease; alloHSCT, allogeneic hematopoietic stem cell transplantation; AML, acute myeloid leukemia; c/oGVHD, chronic/overlap graft-versus-host disease; BW, body weight; DLI, donor lymphocyte infusion; NRM, non-relapse mortality; OS, overall survival; RIC, reduced intensity conditioning. *Disease risk according to ELN [26] and IPSS-R [27].

Table S6A: $CD4^+CD25^+CD127^{low}$ T_{reg} cells

	Expansion	noExpansion	P value
N	9	12	
Sex, male	4 (44)	5 (42)	>0.999
Age at first DLI, years	48 (23-63)	45.5 (33-72)	0.478
Disease details: AML	5 (56)	10 (83)	0.331
Disease risk: adverse*	1 (11)	4 (33)	0.338
Donor age at alloHSCT, years	29 (22-45)	33 (22-64)	0.141
Female donor male recipient	2 (22)	0	0.171
Conditioning regimen: RIC	5 (56)	9 (75)	0.397
aGVHD post alloHSCT	2 (22)	6 (50)	0.367
c/oGVHD post alloHSCT	2 (22)	0	0.171
Antineoplastic treatment pre DLI	4 (44)	8 (67)	0.396
First DLI, months post alloHSCT	9.3 (7.9-24.0)	11.2 (4.6-75.7)	0.464
Dose first DLI, $CD3^+$/kg BW	$0.5x10^7$ ($0.1x10^7$-$1x10^7$)	$0.5x10^7$ ($0.1x10^7$-$2.5x10^7$)	0.507
Total number of DLI	2 (1-4)	2 (1-7)	0.931
Cumulative amount of applied donor cells, $CD3^+$/kg BW	$1x10^7$ ($0.5x10^7$-$16x10^7$)	$1x10^7$ ($0.5x10^7$-$32x10^7$)	0.957
First sampling time point, days post DLI	14 (13-21)	14 (12-18)	0.123
GVHD at first sampling time point post DLI	0	0	>0.999

Table S6B: $CD4^+$ T_{con} cells

	Expansion	noExpansion	P value
N	14	7	
Sex, male	6 (43)	3 (43)	>0.999
Age at first DLI, years	47.5 (23-65)	48 (39-72)	0.582
Disease details: AML	10 (71)	5 (71)	>0.999
Disease risk: adverse*	3 (21)	2 (29)	>0.999
Donor age at alloHSCT, years	33 (22-64)	30 (22-58)	0.502
Female donor male recipient	2 (14)	0	0.533
Conditioning regimen: RIC	8 (57)	6 (86)	0.337
aGVHD post alloHSCT	6 (43)	2 (29)	0.656
c/oGVHD post alloHSCT	1 (9)	1 (14)	>0.999
Antineoplastic treatment pre DLI	8 (57)	4 (57)	>0.999
First DLI, months post alloHSCT	10.0 (4.6-23.8)	24 (5.6-75.7)	0.172
Dose first DLI, $CD3^+$/kg BW	$0.75x10^7$ ($0.1x10^7$-$2.5x10^7$)	$0.5x10^7$ ($0.1x10^7$-$1x10^7$)	0.643
Total number of DLI	2.5 (1-7)	2 (1-3)	0.355
Cumulative amount of applied donor cells, $CD3^+$/kg BW	$2.2x10^7$ ($0.5x10^7$-$32.5x10^7$)	$1x10^7$ ($0.5x10^7$-$6x10^7$)	0.198
First sampling time point, days post DLI	14 (13-21)	14 (12-25)	0.988
GVHD at first sampling time point post DLI	0	0	>0.999

Supplementary Table S7. Patient samples.

All available blood samples are listed for each patient. Abbreviations: DLI, donor lymphocyte infusion; PB, peripheral blood.

Patient ID	Blood samples (days from first DLI)	DLI sample
01	0, 14, 28	yes
02	0, 17, 28	yes
03	0, 13, 27	yes
07	0, 14, 28, 398, 463	yes
08	0, 13, 28	yes
09	0, 18, 33	yes
10	0, 13, 27, 274, 372	yes
11	0, 14, 28, 264, 292, 344, 455, 476, 483, 497	yes
12	0, 14, 28, 223, 273, 343	yes
13	0, 16, 44, 220, 233, 247, 264	yes
16	0, 10, 17, 31, 135, 248, 261	yes
17	0, 21, 27, 105, 125	yes
18	0, 14, 27	no (PB)
19	0, 15, 34, 76	yes
20	0, 21, 41, 112, 136, 164, 240	yes
22	−7, 15, 32, 54, 84, 123	yes
23	0, 8, 14, 43, 64, 91, 117	yes
25	−1, 12, 24, 54, 96, 124	yes
27	0, 13, 33, 77, 99, 126, 191	no (PB)
30	0, 14, 28, 61, 97	yes
33	0, 13, 31, 56, 97, 110	yes

Supplementary Table S8. Healthy control samples.

All available blood samples meeting inclusion criteria are listed for each healthy control. Abbreviations: HC, healthy control.

HC ID	Blood samples (days from study entry)
H16.02	0, 14, 33, 187
H16.03	0, 14, 33, 64, 124, 187
H16.04	0, 14, 124, 187
H16.06	0, 33, 68, 141, 188
H16.07	0, 14, 33, 62, 91, 124, 187
H16.11	0, 14, 30, 65, 93, 119, 183
H16.12	0, 30, 93, 177
H16.13	0, 16, 63, 120
H16.14	0, 13, 28, 63, 90, 121, 208

Supplementary Table S9. Antibody panel for flow cytometry.

	Specificity	Fluorochrome	RRID	Clone	Order number	Company	Dilution
1	CD45	APC-Vio770	AB_2660424	5B1	130-096-609	Miltenyi Biotec	1:100
2	TCRgd	PE	AB_2868642	11F2	333141	BD	1:50
3	TCRab	FITC	AB_2661263	BW242/412	130-098-688	Miltenyi Biotec	1:50
4	CD4	PerCP	AB_2660475	M-T466	130-101-147	Miltenyi Biotec	1:50
5	CD8	VioGreen	AB_2660905	135/80	130-096-902	Miltenyi Biotec	1:50
6	CD25	BV605	AB_244320	4E3	130-091-024	Miltenyi Biotec	1:50
7	CD127	APC	AB_10829303	MB15-18C9	130-094-890	Miltenyi Biotec	1:50
8	Viability	DAPI	n/a	n/a	130-111-570	Miltenyi Biotec	1:200
